# Current knowledge, attitudes and practices of women on breast cancer and mammography at Mulago Hospital

**DOI:** 10.4314/pamj.v5i1.56186

**Published:** 2010-05-06

**Authors:** Kiguli-Malwadde Elsie, Mubuuke A Gonzaga, Businge Francis, Kawooya G Michael, Nakatudde Rebecca, Byanyima K Rosemary, Muyinda Zeridah

**Affiliations:** 1 Radiology department, School of Medicine, College of Health Sciences, Makerere University, Kampala, Uganda; 2 Mulago National Referral Hospital, Kampala, Uganda

**Keywords:** Mammography, Breast cancer, Mulago Hospital, Uganda

## Abstract

**Background:**

Breast cancer is the third commonest cancer in Ugandan women. Women present late for breast cancer management which leads to high mortality rates. The objective of the study was to assess the knowledge, attitudes and practices of Ugandan women concerning breast cancer and mammography.

**Methods:**

This was a descriptive cross-sectional study where 100 women reporting to the Radiology department were interviewed. We used consecutive sampling.  Interviewer-administered questionnaires were used to collect opinions of the participants. For data analysis, answers were described as knowledge, attitude, practice and they were correlated with control variables through the chi-square. Bivariate and logistic regression analyses were also used.

**Results:**

Most of the women (71%) had no idea about mammography. More than 50% did not know about risk factors for breast cancer. The attitude towards mammography was generally negative. Regarding seeking for mammography; level of literacy, occupation and marital status were significant on bivariate analysis, however only level of literacy and employment remained the significant independent variables on logistic regression analysis. The main barrier to mammography was mainly lack of information.

**Conclusion:**

Women in this study had inadequate knowledge and inappropriate practice related to mammography as a procedure for breast cancer investigation.

## Background

Breast cancer is the third commonest cancer in women in Uganda after Kaposi’s sarcoma and cervical cancer [1, 2]. Breast cancer incidence in Uganda is 22: 100,000 [1]. Five year survival rate is 56% [3]. Several studies have reported that breast cancer is the most common cancer and principal cause of cancer deaths in women and is therefore a world concern [4-9]. For example in Brazil, breast cancer is the leading cause of cancer deaths among women [10]. Among Turkish women, breast cancer represents 24.1% of all cancers and is the second leading cause of cancer-related deaths. About 2390 new cases of breast cancer were diagnosed in 1999 in Turkey [11].

Primary randomized controlled trials have shown the importance of mammography in early diagnosis of breast cancer in asymptomatic women and it has been effective in decreasing mortality especially in women aged 50-69 years with reductions of 20% to 35% [12-15]. However, for the women who know about mammography, the costs involved are still very high which prevents them from going for it [16]. Besides the economic issues, other difficulties like fear of irradiation for those who know about it, limited availability of the service, anticipated pain, discomfort and anxiety about mammography also come into play [17, 18]. It has been reported that an annual mammography for women over 50 years of age reduces mortality rate from breast cancer [19-22]. There is a wealth of literature reporting women’s adherence to mammography including their knowledge, behavior and beliefs about breast cancer, knowledge of risk factors, attitudes and mammography [23, 24]. There have been some improvements in encouragement of women to have mammograms. Nevertheless, mammography remains underutilized by women although it can be effective in early detection of breast cancer [24].

The primary factors that increase risk of breast cancer in women include certain inherited genetic mutations, a personal or family history of breast cancer, and biopsy-confirmed hyperplasia [25]. Other factors that increase breast cancer risks include a long menstrual history, obesity after menopause, recent use of oral contraceptives, postmenopausal hormone therapy, nulliparity or having the first child after age of 30, ethnicity characteristics, exposure to radiation, or consumption of one or more alcoholic beverages per day [25, 26]. Factors that decrease breast cancer risks include breastfeeding, physical activity, and the maintenance of a healthy body weight [26, 27]. Unfortunately, many women lack access to all this information. Mammography, Clinical breast examination (CBE) and breast self-examination (BSE) are the secondary preventive methods used for investigation in the early detection of breast cancer [28]. Cancer detection investigations therefore play a pivotal role in reducing breast cancer related mortalities [29]. The American Cancer Society (ACS) recommends CBE and mammography in the early detection of breast cancer [30]. According to ACS recommendations, women should know how their breasts normally feel and report any breast changes promptly to their health care providers. BSE is an option for women starting from the early 20s [31-33]. ACS no longer recommends BSE as there is reliable data that breast cancer detection through BSE does not increase survival rate [30]. However, in a developing and resource-constrained country like Uganda, BSE is an important viable optional substitute, where access to CBE and most importantly mammography is extremely difficult and might still detect breast cancer early enough for treatment which can be offered to prolong women´s lives and reduce suffering [2]. Women in their 20 s and 30 s should have a CBE as part of a periodic health examination by health professionals preferably every 3 years. After the age of 40, women should have a CBE and a mammogram every year, as recommended by the ACS [25].

Zincir reported that the percentage of women over the age of 40 having a professional breast investigation every year was 21.1% in the eastern region of Turkey [34]. Annual mammography is considered the most valuable tool for detecting breast cancer in the earliest possible stages, before the cancer has metastasized and when interventions are most effective, least invasive and debilitating. The decline in breast cancer mortality has been largely attributed to regular mammography investigations [35, 36]. The rate of undergoing a recommended mammography practice was 12.6 % in Secginli´s study performed in Istanbul [37]. The Health Belief Model (HBM) originally introduced in the 1950s has been widely used in health behavior applications including breast cancer investigations [38-41]. The model stipulates that health-related behavior is influenced by a person´s perception of the threat posed by a health problem and by the value associated with his or her action to reduce that threat [42, 43]. According to this therefore, a woman who perceives that she is susceptible to breast cancer and that breast cancer is a serious disease would be more likely to perform regular breast examinations [43,44].

Despite breast cancer being one of the few cancers that can be detected early before seeing symptoms using mammography, mammography is still only performed on a low proportion of the women population in Uganda. Despite the wealth of literature available globally documenting knowledge, attitudes and practices of women about breast cancer and mammography, there is still paucity of literature on the African experience in this area. The aforementioned gaps form the basis of the present study. Hindrances to accessing mammography services not only in Uganda or Africa, but also globally should be identified and then health care authorities should establish strategies to overcome them.

The purpose of this study therefore, was to assess the knowledge, attitudes and practices of women about cancer and mammography as well as identify potential barriers hindering women from accessing mammography services. Hopefully, findings from this study will provide a starting point for health authorities to raise awareness amongst women about breast cancer and the role of mammography in breast cancer investigation. Due to operational costs, this study could neither be extended to all women reporting to the hospital nor to women elsewhere in Uganda. This coupled with no focus group discussions conducted with the study participants are potential limitations of the study. We could not conduct focus group discussions since all women in the study were outpatients and just trickle in the Radiology department it would be difficult to gather them. We suggest more similar studies to be conducted with other groups of women from other areas. Combined with the data from this study, it could provide a better understanding of the barriers faced by women when accessing a procedure such as mammography.

## Methods

The study was conducted at Mulago Hospital which is Uganda´s National Referral Hospital. It also acts as the teaching hospital for the College of Health Sciences, Makerere University. The hospital is located in the northern part of Kampala City, Uganda’s capital and has a bed capacity of 1,500.

It was a descriptive cross-sectional study using interviewer-administered questionnaires. The questionnaires were administered in English and Luganda (a local language) covering the following domains: Demographics (age, marital status, education, employment etc), knowledge, attitudes and practice (KAP) of women on breast cancer and mammography. To ensure validity and reliability of the data collected, the questionnaire was reviewed for information quality and legitimacy and any corrections arising were made. It was then pre-tested before the survey.  Questions asked sought knowledge, attitudes and practices on breast cancer, use of mammography, BSE as well as CBE. Women above 30 years of age reporting to the Radiology department during the study period and had consented were included in the study. Additionally, all women who reported to the radiology department specifically for mammography examination were also excluded because they already had some information about mammography. Correct and consistent answers regarding the value of mammography were considered adequate knowledge. Attitude was considered adequate if women were positive about mammography and supported their statements consistently. Practice was considered satisfactory if women correctly answered questions regarding the use of mammography and the desire of doing it. For women who had not had a mammogram, the main reasons for that were also sought.

100 women reporting to the Radiology department during the study period were involved in the study. Consecutive sampling was used. This was done in order to give an opportunity to every woman who meets the inclusion criteria to participate in the study without any bias.

A quiet place was chosen for the interviews. Responses were compiled, entered in the computer and stored on flash disks and kept securely in a locked place. Only the investigators had access to this information. Information in the computer was protected using passwords.

Data was both quantitative and qualitative. Thematic analysis was used for qualitative data. It involved content analysis to extract the meanings of the participants, and also transcription. Raw data was proof-read and coded into categories of similar meaning. Categories were established, resulting into content themes. These themes summarized the meaning of the data which addressed the purpose of the study. Quantitative data went through cleanliness and consistency tests, frequency analysis and entered into SPSS version 10.0 statistical program. Age of participants was

expressed as means of ± SD. Associations between socio-demographic characteristics and adequacy of KAP of mammography were determined using both bivariate and logistic regression analysis. A Chi-square was used to test statistical significance and a p-value of 0.05 was adopted for this study.

Permission to carry out this study was granted by the Ethics and Research Committee of the College of Health Sciences, Makerere University. Participants provided written consent prior to beginning of the interview after a thorough explanation of what the study was all about by the interviewers. English and Luganda; a local language were used in the consent process, dialects understood by the participants. Verbal consent was obtained from those participants who could not write, also after a thorough explanation. The study strictly followed the principles of the Declaration of Helsinki.

## Results

### Socio-demographic Characteristics

The socio-demographic characteristics of participants are summarized in table 1. From the table, most of the participants belonged to the 4^th^ decade age group. Majority had primary level of education.

### Knowledge

Participants´ knowledge about the incidence and risk factors of breast cancer is provided in table 2. From the table, only few women were aware of the high incidence of breast cancer. More than half of the women did not know about the risk factors for breast cancer. Knowledge about the methods of investigation of breast cancer is summarized in table 3. From the table, majority of the women did not know mammography. The women did not know that X-rays are used during mammography and could not point out any potential risks due to exposure to X-rays.  None of the women responded correctly about breast ultrasound and risk of breast cancer as majority (80%) said that ultrasound has a potential risk of causing cancer. Of the participants, 66 (66%) had done BSE. A significant number of women (40%) had ever had a CBE. However, none of the participants had ever had a mammogram. When each correct answer was scored, the mean score was 42.39. The women who had done BSE had significantly higher scores (p=0.013) than those who had not. The same applies to women who had had a CBE. There was no significant difference in scores between the unemployed and the employed (p=0.602), nor between lower educated participants and those educated beyond primary school level (p=0.821).

### Attitudes

The women’s attitude towards mammography was negative. Most of them confused mammography with breast ultrasound.

*“I can never go back for those checks in machines because I do not see anything”*, one woman commented.

*“Ka T.V ako nkamanyi, naye siyinza kukagendamu mirungi mingi kubanga nyinza okufuna cancer”*, another women retorted, literally meaning that, *“I know about ultrasound, but I cannot go for it frequently as I fear getting cancer”* One lady who had some knowledge about mammography from a friend said:

“I was told that machine presses your breasts so hard causing pain, so I better do BSE and go for CBE instead of going for those other things?”

Majority of the women (62%) said that they were told that they could check their breasts at home monthly and also go for check up with the doctor once in a while.

*“I was told to check my breasts every month by the doctor, but he never told me anything else”*, one lady responded.

### Practices

All women in this study had never had a mammogram. Majority reported that they did not undergo mammography because they had never had of it and its use in investigating breast cancer. The few women who had knowledge about mammography said that they had been told it was expensive and could not afford, hence not seeking for it. Regarding seeking for mammography; level of literacy, occupation and marital status were significant on bivariate analysis (p=0.003), however only level of literacy and employment remained the significant independent variables on logistic regression analysis (p=0.002). Women beyond primary level of education were about four times more likely to go for mammography than women below that level of education (OR 3.79, 95% CI 1.51-9.43). Employed women were seven times more likely than the unemployed to go for mammography, (OR 6.9, 95% CI 1.46-333.21). The impediment to go for mammography expressed by all women was the lack of knowledge about the role of mammography in breast cancer investigation and limited access to mammography services. However, some women had done other investigations like breast ultrasound (12 %). Majority (66%) reported indulging in BSE most frequently. The commonest reason cited for this was that it is convenient and does not cost anything. However, all women who had done breast ultrasound were worried about getting cancer from the scan if they did repeatedly, which also affected the seeking behaviour.

*“I did not go for the scan the last time the doctor told me because I feared getting cancer”*, one lady said.

**Table 1:** Socio-demographic characteristics of participants

**Characteristics**	**Frequency n (%)**
**Age group (mean ± SD)**	`
30-39 (37.1 ± 2.0)	28 (28%)
40-49 (44.9 ± 2.5)	44 (44%)
50-59 (52.9 ± 2.4)	21 (21%)
60-69 (64.2 ± 3.1)	5 (5%)
70-79 (72.5 ± 4.4)	2 (2%)
**Level of education**	
Illiterate	23 (23%)
Primary	60 (60%)
Secondary	12 (12%)
Tertiary	5 (5%)
**Employment**	
Employed	43 (43%)
Unemployed	57 (57%)
**Marital status**	
Married	10 (10%)
Co-habiting	72 (72%)
Single	18 (18%)

**Table 2:** Socio-demographic characteristics of participants

**Items**	**Frequency of participants responding correctly n (%)**
Breast cancer is one of the most common cancers in women	15 (15%)
Breast cancer is a disease with a high incidence of death	10 (10%)
Risk Factors	
Positive correlation with age	20 (20%)
Positive correlation with obesity	13 (13%)
Positive family history of breast cancer	34 (34%)
Presence of previous contra-lateral breast cancer	30 (30%)

**Table 3:** Knowledge about the methods of investigation of breast cancer

**Items**	**Frequency of participants responding correctly n (%)**
**Knowledge about mammography**	
More than sufficient	0 (0%)
Sufficient	4 (4%)
Partially sufficient	25 (25%)
No idea at all	71 (71%)
What type of energy is used during mammography?	0 (0%)
Is there a potential risk of future breast cancer due to energy used in mammography?	0 (0%)
**Knowledge about Ultrasound**	
Have heard about ultrasound	80 (80%)
Used for younger women and a complimentary to mammography	0 (0%)
What type of energy is used in Ultrasound?	0 (0%)
Is there is a potential risk of future breast cancer due to Ultrasound?	0 (0%)
	
Mammography can show early breast cancer	2 (2%)
Healthy women should have control mammography at certain intervals	15 (15%)
There is an age limit for mammography	10 (10%)
Most appropriate age group (35-45) for first mammogram	0 (0%)

## Discussion

### Knowledge

The 3 methods recommended for detection of breast cancer are BSE, CBE by a health care professional, and mammography, the third being the most effective [2,5,7,20]. A consortium of American medical organizations, including the American Cancer Society, has issued the following recommendation: between the ages of 40 and 49 years, women should undergo a CBE and mammography every year or 2. Women older than 50 years should have an annual CBE as well as a mammogram [4, 7, 20]. This study showed that majority of the women were informed about BSE as well as CBE, but little about mammography. This could be explained by the limited mammography services in Uganda. Mammography can only be accessed in the National referral hospital and a few private health facilities found in the capital city which leaves the majority of women and even health workers in rural areas ignorant about it. This finding is indeed similar to what Aylin et al found in their study where majority of the women had no idea about mammography [45].

This study also showed that majority of the women did not know about risk factors for breast cancer. The reason for this is again due to lack of exposure to knowledge and facts about breast cancer. The low levels of education in majority of the women who participated in this study is another reason, in that the women may not know how to access accurate information about breast cancer. Like Aylin et al [45] found in an earlier study, this study also showed that majority of the women did not know the age range when mammography should be done nor did they know its potential in detecting early breast cancer. Regarding breast ultrasound, majority of the women thought that it is a potential risk for future breast cancer. This striking finding is contrary to what Aylin et al [45] found out, but it is similar to what Mubuuke et al found in an almost similar population [46]. This is most likely due to lack of knowledge about ultrasound. It also calls for a comprehensive Ministry of Health program to educate women about ultrasound so that such misconceptions are cleared.

### Attitudes

All women generally reported a negative attitude towards mammography. The limited knowledge about mammography probably contributed to the negative attitude since they did not know its exact role. However, the participants expressed willingness to change their attitude towards mammography provided they got adequate information regarding its role. This is partly due to the wide campaign about breast cancer and its effects not only in Uganda, but globally as well. Therefore, being an investigative procedure to detect breast cancer is probably the main reason for women having acceptance for it provided they get to know its benefits. Majority of women (75%) in this study feared that breast ultrasound would pose a potential risk to cancer. Conversely, Aylin et al found out in their study that majority of the women (72.5%) were comfortable with breast ultrasound without any fear [45]. This shows that the women in Uganda and probably in many developing societies confuse breast ultrasound with mammography. In support of these findings, an earlier study done by Mubuuke et al (46) again with Ugandan women showed that women think ultrasound can cause cancer. This unveils a huge knowledge gap as far as breast ultrasound is concerned probably in all developing societies and therefore calls for more sensitization and education of society regarding the issues raised by the health workers.

Some women who had done CBE reported embarrassment especially when being examined by a male doctor which changed their attitude towards breast screening procedures. This finding was also reported by Dibble et al [4] and Saint-Germain [20]. Although women in this study had not done any mammography, the procedure of carrying out the mammogram may turn out to be embarrassing as well. Women in the Ugandan society have a much closed culture and a traditional, conservative and male-dominated life style. The theory of reasoned action suggests that the intention to participate in mammography for breast cancer is determined primarily by 2 factors: the woman’s attitude toward the procedures and the social normative influence of the people, who are important in her life, most notably spouses [4]. Educational programs and sensitization campaigns about mammography can be accomplished in a culturally sensitive manner by considering these points. Husbands should be included in the breast cancer sensitization programs. They cannot only support their wives and prevail on them to change their attitude towards mammography, but can also reduce their own risk of breast cancer morbidity and mortality [8, 24].

### Practices

Majority of the women frequently practiced BSE and occasionally sought for CBE, but did not go for mammography. It is thought that BSE makes women more aware of their breasts which in turn may lead to an earlier diagnosis of breast cancer [21]. The rationale behind extending BSE practice as a screening test is the fact that breast cancer is frequently detected by women themselves without any other symptoms. A metaanalysis of studies investigating the possible benefits of BSE has shown that regular practice increases the probability of detecting breast cancer at an early stage [5]. This study revealed the finding that many participants had practiced BSE. Most of the women in this study were from diverse backgrounds and mainly from lower social status. This means that these women may not have ready access to mammography and CBE. In their study, Siahpush & Singh also reported a similar finding with women from non-metropolitan backgrounds [21]. ACS no longer recommends BSE (30). However, in developing societies like Uganda, BSE should still be encouraged because access to CBE and most importantly mammography is extremely limited. Some health facilities are not easily accessible and mammography is very expensive for the majority, yet BSE can still help to some extent.

There may be several reasons for not undergoing mammography. The cost of mammography in Uganda and probably globally is high, particularly for a woman who does not have social security like most of the participants in this study. Although Dibble et al [4] have reported factors like mammography-induced pain and discomfort plus the effects of the radiation received during a mammogram, as a barrier, this cannot apply to this study as all the women interviewed had not undergone any single mammogram. This means that there is something more than pain or the fear of radiation that hinders these women from seeking mammography. The lack of information about mammography and the high costs for the few who know about it may be the biggest hindering factors especially in low-resourced settings. Focused educational programs are urgently needed to address this issue. Programs for women, especially those who have low education levels, do not work and spend most of their time at home, should be encouraged. For this purpose, the media (local written and oral, radio, television, soap operas, newspapers etc.) could be used. Through such programs, awareness of breast cancer, the importance of its early diagnosis, and prompt treatment can significantly increase.

## Conclusion

Participants in this study lacked adequate knowledge had poor attitude and inappropriate practice about mammography. The main barrier to mammography was lack of information about its role in early detection of breast cancer which improves survival. Awareness campaigns and subsidizing the costs for mammography by Ministry of Health would improve survival from breast cancer. Involving men as well in this awareness will also greatly improve the current situation. All this should be coupled with acquisition of mammography machines by regional referral hospitals to ease access to the service.

## Competing interests

The authors declared that they have no competing interests
